# The relationship between depression risk perception and self-help behaviours in high risk Canadians: a cross-sectional study

**DOI:** 10.1186/s12889-020-08983-0

**Published:** 2020-06-06

**Authors:** Emily Warner, Molly Nannarone, Rachel Smail-Crevier, Douglas Manuel, Bonnie Lashewicz, Scott Patten, Norbert Schmitz, Glenda MacQueen, Jian Li Wang

**Affiliations:** 1 The Institute of Mental Health Research, University of Ottawa, Ottawa, Canada; 2 School of Epidemiology and Public Health, Faculty of Medicine, University of Ottawa, Ottawa, Canada; 3 Clinical Epidemiology Program, Ottawa Hospital Research Institute, Ottawa, Canada; 4 Department of Family Medicine, Faculty of Medicine, University of Ottawa, Ottawa, Canada; 5 Department of Community Health Sciences, Cumming School of Medicine, University of Calgary, Calgary, Canada; 6 Department of Psychiatry, Cumming School of Medicine, University of Calgary, Calgary, Canada; 7 Department of Psychiatry, Faculty of Medicine, McGill University, Montreal, Canada

**Keywords:** Risk perception accuracy, Self-help behaviours, Major depressive episode

## Abstract

**Background:**

Self-help may reduce the risk of depression, and risk perception of depression may influence initiating self-help. It is unknown how risk perception is associated with self-help behaviours. The objectives of this study are to (1) describe the self-help strategies used by high-risk Canadians in relation to the accuracy of perceived depression risk, by sex, and (2) identify demographic and clinical factors associated with self-help behaviours.

**Methods:**

Baseline data from a randomized controlled trial including 358 men and 356 women at high-risk of developing depression were used. Following methods used in cancer research, risk perception accuracy was determined by comparing the participant’s self-perceived and objective risk of developing depression and classifying as accurate, over-estimation and under-estimation based on a ± 10% threshold. The participant’s objective depression risk was assessed using sex-specific multivariable risk predictive algorithms. Frequency of using 14 self-help strategies was assessed. One-way ANOVA testing was used to detect if differences in risk perception accuracy groups existed, stratified by sex. Linear regression was used to investigate the clinical and demographic factors associated with self-help behaviours, also stratified.

**Results:**

Compared to accurate-estimators, male over-estimators were less likely to “leave the house daily,” and “participate in activities they enjoy.” Male under-estimators were also less likely to “participate in activities they enjoy.” Both male ‘inaccurate’ perception groups were more likely to ‘create lists of strategies which have worked for feelings of depression in the past and use them’. There were no significant differences between self-help behaviours and risk perception accuracy in women. Regression modeling showed negative relationships between self-rated health and self-help scores, irrespective of sex. In women, self-help score was positively associated with age and educational attainment, and negatively associated with perceived risk. In men, a positive relationship with unemployment was also seen.

**Conclusions:**

Sex differences exist in the factors associated with self-help. Risk perception accuracy, work status, and self-rated health is associated with self-help behaviours in high-risk men. In women, factors related to self-help included age, education, self-rated health status, and perceived risk. More research is needed to replicate findings.

**Trial registration:**

Prospectively registered at ClinicalTrials.gov (NCT02943876) as of 10/21/16.

## Background

Major depressive disorder (MDD) is a prevalent and disabling mental illness. A 2012 study by Patten and colleagues [1] found that the annual prevalence of MDD in Canada was 3.9%, with women and younger age groups experiencing a higher prevalence. Individuals who suffer from MDD are at an increased risk of developing other mental health disorders, such as anxiety disorders or substance dependence, as well as physical conditions such as heart conditions and diabetes [2]. MDD can re-occur throughout the lifespan, and even increase one’s risk of mortality, especially in cases of comorbidity with other mental and physical health conditions [1]. Due to these factors, MDD inflicts a significant burden of disease on society by increasing healthcare costs and impairing the workforce productivity [3].

Given the severe and potentially lifelong implications that the development of MDD can have on an individual, exploration into MDD prevention is gaining momentum in the field of mental health research. MDD has a multi-factorial etiology and can be a result of the interaction between demographic, biological, clinical and psychosocial factors (e.g., exposure to negative life events, stressors, etc.). Self-help strategies are commonly used to deal with stress and milder forms of depression through reduced exposure to risk factors and enhanced resilience and self-efficacy. Many prevention strategies have been proposed in the prevention and treatment of MDD, including effective self-help techniques [4, 5]. The use of informal self-help techniques for populations experiencing symptoms of depression and anxiety and for individuals who are at high risk has the potential to aid in early intervention, boost a sense of self-efficacy, and create easily accessible care for individuals who perceive stigma as a barrier to formal mental healthcare [6, 7]. Researchers believe that promoting effective, evidence-based self-help strategies to individuals may help reduce the disease burden associated with MDD through early prevention by providing easily accessible and economical supplemental option to formal professional intervention [6]. Through an international Delphi study, Morgan and Jorm identified 14 self-help strategies that are perceived to be effective and easy to implement, including strategies related to sleep hygiene, diet, and exercise [7].

According to the Health Belief Model [8], the way an individual perceives their personal risk of developing an illness (such as MDD) can influence their health behaviours [9], such as help seeking and self-help behaviours. For example if an individual does not perceive their risk of developing an illness as high, they would display less help-seeking behaviour and would be less likely to follow-through with intentional self-help techniques. Currently, there is a paucity of research on the relationship between perceived depression risk and self-help behaviours.

An individual’s accuracy of risk perception is based on their perceived risk of illness development in comparison to one’s objective risk. Objective risk is often determined through multivariable risk predictive algorithm (MVRP) which determines the probability of developing a specific condition over a given timeframe [10, 11]. For example, the objective risk of having a heart disease over 10 years is commonly estimated by the Framingham risk score [11]; the objective risk of having breast cancer is often estimated using the Gail model [10]. The discrepancy between one’s perceived risk and objective risk may lead to three outcomes; accurate, overestimate or underestimate (i.e., inaccurate), all of which may influence changes in health behaviour and outcomes [9]. An individual who underestimates their risk of developing a specific disease could potentially not engage in self-help behaviours, thinking the actions are inconsequential due to perceived low risk [12]. Overestimating one’s risk is not necessarily healthier in terms of health outcomes, as it may lead to enhanced stress, unnecessary health service use, as well as a potentially exhibiting ‘helpless’ behaviour wherein an individual believes that there is nothing that they can do to better their situation [13] and correspondingly, does not participate in self-help behaviours. While research has shown that people are more likely to underestimate their risk of developing physical illnesses (e.g., heart disease) [14], little information is known about accuracy of risk perception of MDD, as well as how this risk perception reflects in the use of self-help behaviours. The objectives of this study are to (1) describe the self-help strategies used by high risk Canadians in relation to the accuracy levels of their perceived depression risk, relative to sex, and (2) identify demographic, socioeconomic, and clinical factors associated with self-help behaviours.

## Methods

For the objectives of this study, we used data from an ongoing randomized controlled trial (RCT) in men and women who are at high risk of having a major depressive episode (MDE). The RCT sought to investigate whether: (1) the disclosure of personalized depression risk information (determined by the sex-specific multivariate risk predictors (MVRPs)) encouraged high-risk individuals to take preventive actions; and (2) whether the mental health status of the high-risk individuals was negatively impacted by the disclosure of personalized depression risk information. Detailed information about study methodology, design, data collection, and sample size calculation can be found in Wang et al. (2019) [15]. This study was prospectively registered at ClinicalTrials.gov (NCT02943876) as of October 21st, 2016.

The RCT was conducted in men and women separately, and had a one-to-one intervention-to-control group ratio, in which the intervention arm received their personalized risk of developing MDE. The target population of the RCT are individuals in the community who are at high risk of MDE. Individuals included in this study met the following inclusion criteria:

- Were a minimum of 18 years of age,
- Did not have a history of MDE in the 12 months before being interviewed or were in remission from MDE symptoms at the time of the interview, as determined by the following criteria,
- Were deemed to be at high risk of MDE, as determined by the sex-specific MVRPs

(men = 6.5%+; women = 11.2%),

- Available for 6 and 12 month follow-up assessments
- Fluent in either English or French

Participants were classified as ‘in remission’ based on their response to the question: “In the past 2 months or longer, has your mood been much improved or back to normal AND you DIDN’T have the symptoms of?” The inspiration for this question was from the US National Epidemiological Survey on Alcohol and Related Conditions [16].

### Recruitment

Participants for the RCT were recruited using the random digit dialing method from January 2018 until February 2019. A telephone survey firm with access to landline and cell phone numbers nationally completed the screening, baseline assessment, and randomization stages of the RCT. In the recruitment process, 95,948 phone numbers were dialled; 11,753 of which were not valid, 2683 did not meet eligibility criteria, 80,795 did not complete the baseline interview. Overall, 714 participants (358 men, 356 women) were recruited and randomly assigned to either the control or intervention groups. Ethics approval for this study was given by the Royal’s Research Ethics Board.

### Measurements

*Personalized risk of having MDE* was estimated by sex-specific risk predictive algorithms (MVRP) for MDE. Detailed information about the development of the MVRPs are described in a previous publication [17]. The MVRPs were developed, using longitudinal data from 4737 men and 5846 women who were randomly selected across Canada, and who had not had a MDE in the past year prior to the baseline. A participants probability (personalized risk) of having MDE over the next 4 years was determined by participants responses to a series of questions about family and personal history of MDE, and life stress and childhood traumatic experience [17]. The discriminative power of the MVRPs are good and consistent with C-statistic algorithms seen in cardiological research, as well as having exceptional calibration [17, 18]. In men, the observed 4-year risk was 5.15% and the predicted 4-year risk was 5.25% for developing a MDE. In women, the observed and predicted 4-year risk was 8.47 and 8.31% for the development of a MDE, respectively [17]. The top 2 deciles were used as upper-limit cut-off points, in which 6.5% was set at the ‘high-risk’ cut-off for men and 11.1% was set as the ‘high-risk’ cut-off point for women. The MVRPs was validated in Canadians belonging to a variety of sub-populations as well (rural vs. urban, white vs non-white, immigrants vs non-immigrants) [17].

*Perceived depression risk* was evaluated by asking the following question: “How likely are you to get depression in the next 4 years?” on a range of 0–100 in which 0 indicated depression was impossible to occur, and 100 indicated they were certain it would occur [19, 20].

*Accuracy of risk perception* was determined by subtracting the participants perceived depression risk from their individualized risk of developing depression [19, 20], as determined by the MVRP’s algorithm [17]. Positive values of the difference (D) indicate overestimation of risk; negative values indicate underestimation. The same approach as Rimer et al. [19] and Lerman et al. [20] in order to determine the accuracy of depression risk perception. Three categorical variables were derived from this difference: underestimation (<− 10%), overestimation (> 10%), and accurate (− 10% < D < 10%).

*Self-help behaviours* were measured by the Self-help Management Strategy Use Scale (SSUS) developed by Morgan and Jorm [7]. The SSUS assesses the frequency of using each of 14 self-help strategies, rated on a 5-category scale. The SSUS has good internal consistency in this study (Cronbach’s a = 0.78), which is consistent with the alpha value of development (Cronbach’s a = 0.80) [20]. Self-help behaviours were scored on a scale of 0–4 per behaviour, with higher scores indicating increased frequency of participation of said self-help behaviours. Total self-help scores were determined by adding up the score of each individual self-help behaviour. Data was excluded for participants who responded, “I don’t know” to any self-help behaviour. Overall, self-help total scores could range from 0 to 42, with a higher score indicating more frequent use of self-help strategies.

*Other measures* included demographic (sex, marital status, age), socioeconomic (education, employment) and clinical (history of MDD in self or family, self-rated health, mental health service use, and non-specific psychological stress) information. Non-specific psychological distress, as measured by the 10-item version of the non-specific psychological distress scale (K10). The K10 was designed to yield a global measure of distress based on questions about anxiety and depressive symptoms experienced in the last 4-weeks, and has been seen to strongly discriminate between cases and non-cases of DSM-IV classified disorders [21, 21]. In this study, possible K10 scores ranged from 10 to 50, with a higher score indicating more severe psychological distress. Self-rated health was assessed via phone interview by asking the participant “In general, would you say your health is …” with possible responses of excellent, very good, good, fair, and poor. Mental health service use was evaluated with the question “In the past 12 months, that is, from <<OneYearAgo>> to yesterday, have you seen or talked on the telephone with a health professional about your emotional or mental health?”

### Statistical analysis

Statistical analysis for this study was completed using STATA (Version 14) [22]. All analyses were conducted separately in men and women as the MVRP algorithms to determine predicted risk of MDE (%) are sex-specific. Using the chi square test (alpha = 0.05), we estimated and compared the proportions of using 14 self-help strategies between accurate (reference group), overestimation and underestimation groups, with a significance level of 0.005 for Bonferroni correction.

Normality of the distribution of self-help scores was evaluated using the skewness and kurtosis of the residuals, as well as the Shapiro-Wilk normality test (alpha = 0.05). In the event of non-normal distributions, the BoxCox transformation was used in order to determine the coefficient which was used to normalize the data and the appropriate transformation was determined through trial-and-error.

A one-way analysis of variance (ANOVA) was performed to compare the mean total self-help scores amongst the three risk-perception groups. The three risk-perception groups were then compressed into two groups, in which over-estimators and under-estimators were grouped into an “inaccurate perception” group and compared to the “accurate perception” group via a two-sample t-test of means.

We examined the bivariate relationship between self-help score and selected variables. Factors investigated included accuracy of risk perception (accurate vs inaccurate risk perception), age (categorical), income level (categorical), marital status (categorical), highest educational level achieved (categorical), work status (categorical), K10 scores (continuous), speaking to a mental health professional over the last year (categorical), self-rated health (categorical), and perceived risk and predicted risk (continuous) of developing an MDE. A multivariable linear regression model was used to identify the demographic, socioeconomic, and clinical characteristics associated with self-help behaviour in men and women. The model was created using backward-step selection. We first included all variables that were significant in the bivariate analysis in the model. Variables that were not statistically significant (*p* > 0.05) were removed from the model.

The assumptions of linear regression were evaluated utilizing a variety of tests. We assessed the normality of the distribution of self-help scores (dependent variable) by examining the skewness and kurtosis of self-help score residuals, as well as the Shapiro-Wilk normality test (alpha = 0.05). Both q-plots and p-plots of the self-help residual scores were used to visually evaluate the assumption of normality. The assumption of homoscedasticity was evaluated using the Breusch-Pagan / Cook-Weisberg test for heteroscedasticity (alpha = 0.05), as well as visually evaluating the residual plots of self-help scores. The assumption of collinearity was evaluated using the correlation coefficients between independent variables of each model and the variance inflation factor (VIF).

## Results

The demographic, socioeconomic and clinical characteristics of high-risk men and women are presented in Table 1. Overall, 15 men and 19 women did not provide information about their perceived risk of developing major depression and were therefore excluded from the analysis, resulting in a total of 343 men and 337 women being included. It was found that 29.7% of men and 21.7% of women of the sample accurately perceived their risk of developing major depression, while 47.5% of men and 59.6% of women overestimated their risk, and 22.8 and 18.7% underestimated their risk respectively.

**Table 1:** The demographic, socioeconomic, and clinical characteristics of participants at high risk of a major depressive episode

Variables	Men n (%)	Women n (%)
**Gender**	358 (50.14)	356 (49.86)
**Age Group**		
18-30 yrs	102 (28.5)	44 (12.4)
31-50 yrs	128 (35.8)	124 (34.8)
51-64 yrs.	77 (21.4)	114 (32.0)
65 + yrs	51 (14.3)	74 (20.8)
**Marriage Group**		
Married/Common Law	177 (49.6)	213 (60.2)
Single	135 (37.8)	207 (20.3)
D/S/W	45 (12.6)	69 (19.5)
**Work**		
Working for pay	226 (63.1)	184 (52.0)
Not working	132 (36.9)	170 (48.0)
**Education**		
< High-school	42 (11.8)	35 (9.9)
High-school	97 (27.2)	77 (21.7)
Post Secondary	217 (61.0)	243 (68.4)
**Region**		
BC/AB/SK/MB	101 (28.2)	127 (35.7)
ON/QB	220 (61.5)	196 (55.1)
NB/NS/PEI/NL/NT/NV	37 (10.3)	33 (92)
**Self-Rated Health**		
Excellent	33 (9.2)	26 (7.3)
Very good/good	223 (62.3)	206 (57.9)
Fair/poor	102 (29.5)	124 (34.8)
**MDE in the Past Year**		
Yes	65 (18.2)	84 (23.6)
No	293 (81.8)	272 (76.4)
**MDE Before Last Year**		
Yes	17 (4.8)	36 (10.1)
No	341 (95.2)	320 (89.9)
**Psychological distress (K10) (median)**	20	21
**Predicted risk of MDE (median)**	16.07	24.015
**Talked to health professional about Mental Health problems in the past year**		
Yes	88 (24.86)	106 (29.78)
No	266 (75.14)	250 (70.22)

We estimated the proportions of frequent use of each self-help behaviour by levels of accuracy of risk perception (see Table 2). There were no significant differences in using the individual self-help behaviours in women after the Bonferroni correction. The data showed that men in the ‘overestimation’ and ‘under-estimation’ categories differed in several individual self-help behaviours, compared to men in the “accurate” group. Men in the “over-estimate” group were less likely to ‘make sure [they] got out of the house for at least a short time each day’, in comparison to the reference group. Men in both ‘inaccurate’ categories were less likely to “do something [they] enjoy” than the reference group. Men in the ‘inaccurate’ categories were more likely to “[make] a list of strategies that have worked in the past for depression and use them’.

**Table 2:** Self-help behaviours associated with accuracy of depression risk perception in high risk men (*n* = 358)

Behaviours	Accurate (reference) n(%)	Over-Estimation n(%)	Under-Estimation n(%)
You made sure you got out of the house for at least a short time each day			
- not at all/infrequently	16 (15.8)	44 (27.0)*	16 (20.8)
- moderately/very frequently	85 (84.2)	**119 (73.0)**	61 (79.2)
You tried to remain involved in purposeful activities for at least a small part of every day			
- not at all/infrequently	22 (21.6)	46 (28.2)	13 (16.9)
- moderately/very frequently	80 (78.4)	117 (71.8)	64 (83.1)
You rewarded yourself for reaching a small goal			
- not at all/infrequently	49 (48.0)	72 (44.7)	30 (39.0)
- moderately/very frequently	53 (52.0)	89 (55.3)	47 (61.0)
You ate a healthy, balanced diet			
- not at all/infrequently	19 (18.6)	38 (23.5)	14 (18.0)
- moderately/very frequently	83 (81.4)	124 (76.5)	64 (82.0)
You got enough sleep at night and had a bed time and rising time that varied little from day to day			
- not at all/infrequently	40 (40.0)	67 (41.4)	25 (32.5)
- moderately/very frequently	59 (60.0)	95 (58.6)	52 (67.5)
You tried methods to improve your sleep			
- not at all/infrequently	46 (45.5)	72 (45.0)	40 (52.0)
- moderately/very frequently	55 (54.5)	88 (55.0)	37 (48.0)
You did something you enjoy			
- not at all/infrequently	7 (6.9)	34 (21.0)**	17 (18.0)*
- moderately/very frequently	95 (93.1)	**128 (79.0)**	**64 (82.0)**
You engaged in an activity that gave you a feeling of achievement			
- not at all/infrequently	21 (20.8)	47 (29.0)	18 (23.4)
- moderately/very frequently	80 (79.2)	115 (71.0)	59 (76.6)
You talked over problems or feelings with someone who is supportive and caring			
- not at all/infrequently	34 (33.3)	56 (34.6)	19 (24.4)
- moderately/very frequently	68 (66.7)	106 (65.4)	59 (75.6)
You engaged in exercise or physical activity			
- not at all/infrequently	28 (27.5)	52 (31.9)	22 (28.2)
- moderately/very frequently	74 (72.5)	111 (68.1)	56 (71.8)
You made a list of strategies that have worked in the past for depression and used them			
- not at all/infrequently	81 (80.2)	107 (66.5)*	43 (56.6)**
- moderately/very frequently	20 (19.8)	**54 (33.5)**	**33 (43.4)**
You let family and friends know how you are feeling			
- not at all/infrequently	47 (46.1)	79 (48.5)	28 (35.9)
- moderately/very frequently	55 (53.9)	84 (51.5)	50 (64.1)
You enlisted a trusted friend or relative to help you get out and do activities			
- not at all/infrequently	55 (53.9)	89 (54.9)	42 (53.9)
- moderately/very frequently	47 (46.1)	73 (45.1)	36 (46.1)
You have learned relaxation methods			
- not at all/infrequently	56 (54.9)	86 (53.4)	32 (41.6)
- moderately/very frequently	46 (45.1)	75 (46.6)	45 (58.4)

* Significant at a level of 0.05

** Significant at a level of 0.1

The self-help scores in female participants showed a normal distribution based on the Shapiro-Wilk W test for normality (*p* = 0.572). The male population was found, however, to not have a normal distribution of self-help scores (*p* = 0.0012). The BoxCox transformation was used to determine the coefficient which would normalize this distribution in the male population. All male self-help scores were then raised to the power of the coefficient of 1.328811, which resulted in a normal distribution of the total self-help frequencies, as according to the Shapiro-Wilk test (*p* = 0.315). Moving forward in the statistical analysis, the raw female total self-help scores were used, however for the male population, the transformed self-help values were used.

We compared the mean total self-help scores by the three levels of risk perception (see Table 3). There were significant differences between the mean self-help scores of the three risk perception groups in men (*p* = 0.05). There were no significant differences between the means of the risk perception groups in women (*p* = 0.57). Combining the “underestimation” and “overestimation” into one group (“inaccurate”), the data showed that there was no significant difference in self-help scores between the groups in men or women.

**Table 3:** Summary and One-Way ANOVA Data of Self-Help Scores in risk Perception Groups in Men and Women at High Risk of Developing MDE

Gender		Accurate	Underestimate	Overestimate	Total
**Women (*n*** **= 314)**	**Mean**	24.1	24.5	23.5	23.8
	**Standard Deviation**	7.0	7.0	7.0	7.0
	**Df (Between Groups)**	2			315
	**Df (Within Groups)**	313			
	**F (Between Groups)**	0.6			
	**Prob > F (Between Groups)**	0.6			
**Men (*n*** **= 318)**	**Mean**	24.3 (71.2)^a^	25.7 (76.2)^a^	23.4 (67.0) ^a^	24.2 (70.0)^a^
	**Standard Deviation**	7.8 (2.8)^a^	7.7 (3.4)^a^	6.6 (2.0)^a^	7.3 (1.5)^a^
	**Df (Between Groups)**	2			319
	**Df (Within Groups)**	317			
	**F Between Groups**	3.0^b^			
	**Prob > F (Between Groups)**	0.05^b^			

^a^Male data presented in brackets is result of normalization transformation

^b^F values in male data presented are result of normalization transformation

We examined the bivariate relationship between self-help score and selected variables (Table 4). The data showed that, in male participants, perceived risk, unemployed work status, and fair/poor self-rated health were significantly associated with self-help scores; in female participants perceived risk, predicted risk, age, self-rated health, and educational attainment were significantly associated with self-help scores.

**Table 4:** Factors associated with self-help behaviour in men (*n* = 318) and women (*n* = 316) at high risk of developing a MDE: a bivariate analysis

Gender	Variables	Regression Coefficient	Confidence Interval (95%)	***P***-Value
**Males**	**Perceived Risk**	**−0.1**	**−0.2, −0.02**	**0.0**
	Predicted Risk	0.0	−0.2, − 0.1	0.8
	Accuracy Level			
	Accurate	–	–	–
	Underestimate	5.1	−3.1, 13.2	0.2
	Overestimate	−4.1	−10.9, 2.7	0.2
	Age			
	18 - 30 yrs	–	–	–
	31 - 50 yrs	−1.1	−8.3, 6.2	0.8
	51 - 64 yrs	0.4	−7.8, 8.7	0.9
	65 + yrs	5.0	−4.4, 14.5	0.3
	Talked to a Mental Health Professional	−1.7	−8.3, 5.0	0.6
	Education			
	< High School	–	–	–
	High School	−5.1	−15.3, 5.1	0.3
	Post Secondary	−2.5	−11.8, 6.8	0.6
	Marital Status			
	Single	–	–	–
	Married/ Common Law	1.6	−7.9, 4.6	0.6
	D/S/W	−1.9	−11.4, 7.5	0.7
	**Work Status**			
	Working	–	–	–
	**Not Working**	**7.0**	**1.1, 12.9**	**0.0**
	**Self-Rated Health Status**			
	Excellent	*–*	–	–
	Very Good/ Good	6.4	−16.5, 3.6	0.2
	** Fair/ Poor**	**−11.6**	−**22.5, −0.8**	**0.0**
**Females**	**Perceived Risk**	**−0.1**	**−0.1, −0.0**	**0.0**
	**Predicted Risk**	**− 0.1**	**− 0.2, 0.1**	**0.0**
	Accuracy Level			
	Accurate	–	–	–
	Underestimate	−0.7	−3.1, 1.8	0.6
	Overestimate	2.2	−0.1, 4.5	0.1
	**Age**			
	18 - 30 yrs	–	–	–
	**31 - 50 yrs**	**2.6**	**0.1, 5.1**	**0.0**
	51 - 64 yrs	2.4	−0.2, 4.9	0.1
	**65 + yrs**	**3.4**	**0.6, 6.2**	**0.0**
	Talked to a Mental Health Professional	−1.2	−2.8, 0.5	0.2
	**Education**			
	< High School	–	–	–
	High School	2.6	- 0.3, 5.5	0.1
	** Post Secondary**	**4.8**	**2.2, 7.4**	**0.0**
	Marital Status			
	Single	–	–	–
	Married/ Common Law	0.7	−1.3, 2.7	0.5
	D/S/W	1.4	−1.1, 3.8	0.3
	Work Status			
	Working	–	–	–
	Not Working	−0.5	−2.0, 1.0	0.5
	**Self-Rated Health Status**			
	Excellent	*–*	–	–
	Very Good/ Good	−2.9	−5.8, 0.0	0.1
	** Fair/ Poor**	**−6.0**	**−9.0, −3.0**	**0.0**

We included the significant variables in bivariate analysis into multivariate linear regression modeling. In men, the final linear regression modeling showed negative associations between self-rated health status (using ‘excellent’ self-rated health as reference) and the self-help score. It also showed a positive relationship between not working (using ‘working’ as a reference) and total self-help score. Perceived risk initially was seen to be significant in a negative relationship with self-help score in bivariate analysis, however appears to have been confounded by self-rated health (*p* = 0.1), therefore perceived risk was also kept in this model (Table 5).

**Table 5:** Factors associated with self-help behaviour in men (*n* = 318) and women (*n* = 316) at high risk of developing a MDE: a linear regression model

Gender	Variables	Regression Coefficient	Confidence Interval (95%)	P
Men	Perceived risk	−0.1	−0.2, 0.0	0.1
	**Self-Rated health**			
	Excellent (Reference)	–	–	–
	Very good/good	−7.9	−18.3, 2.5	0.1
	^**a**^**Fair/poor**	**−14.8**	**−26.6, −3.1**	**0.01**
	**Work Status**			
	Working (Reference)	–	–	–
	^**a**^**Not Working**	**8.0**	**1.9, 14.2**	**0.01**
Women	**Perceived risk**	**0.0**	**−0.1, 0.0**	**0.0**
	Predicted Risk	0.0	−0.1, 0.0	0.1
	**Age**			
	18 - 30 yrs	–	–	–
	31 - 50 yrs	2.1	−0.2, 4.5	0.1
	**51 - 64 yrs**	**2.8**	**0.4, 5.2**	**0.0**
	**65 + yrs**	**3.1**	**0.4, 5.8**	**0.0**
	**Self-Rated Health**			
	Excellent (Reference)	–	–	–
	Very good/good	−1.9	−4.8, 0.9	0.2
	^**a**^**Fair/poor**	**−4.3**	**−7.5, −1.2**	**0.0**
	**Education**			
	< High School (Reference)	**–**	–	–
	High School	1.3	−1.5, 4.1	0.4
	^**a**^**Post Secondary**	**3.8**	**1.3, 6.3**	**0.0**

^a^Significant at a 0.05 level

In women, significant associations were found between total self-help score and age, self-rated health, perceived risk of having MDE, and education. Perceived risk was found to have a negative relationship with self-help score, as did having a self-rated health status of fair/poor. Both age and education were seen to have a positive relationship with self-help scores in women. Predicted risk was seen to be significantly related to self-help in women, but not men, in bivariate analysis, however it appears to have been confounded by self-rated health in the multiple regression, therefore it was kept in the model (*p* = 0.1) (Table 5). Accuracy of risk perception was not associated with self-help score in the multivariate linear regression models in both men and women.

## Discussion

In this study of men and women who were at high risk of having major depression, we found self-help behaviours differed by accuracy level of depression risk perception in men, but not in women. Compared to men who had an accurate risk-perception, men who were over-estimators were less likely to “get out of the house every day”, and less likely to “do something [they] enjoy.” In both over-estimating and under-estimating men we also saw an increased likelihood of making “lists of strategies which have worked in the past for depression and used them”, in comparison to men with accurate risk perceptions. In both men and women, individuals who rated their health as poor (compared to individuals who rated their health as excellent), were less likely to use self-help strategies. In high risk women, self-help score was significantly associated with age, and educational attainment.

There has not been any previous research on accuracy of risk perception and self-help behaviour in individuals at high risk of developing an MDE. Therefore, we cannot make direct comparison with previous research. Self-help strategies have the potential to be an effective, cost-efficient, preventative method to support individuals at high risk of developing major depression [7]. An individual’s perception of their risk of developing MDE and the accuracy of risk perception may influence his/her participation in self-help strategies [9]. Research has shown that individuals who over-estimate their risk may be subjected to increased levels of stress and worry [9]. Individuals who under-estimate their risk may not feel the need to participate in self-help behaviours [9].

We found that men who over estimate their depression were less likely to “get out of the house every day”, and less likely to “do something [they] enjoy.” Individuals who suffer from depression often display behaviours of learned helplessness, which is the tendency to feel as if they are unable to change the outcomes of the events around them [23]. It is possible that the lack of doing things one enjoys, or leaving the house daily to improve mood in this sub-group may be a by-product of this learned-helplessness wherein individuals may believe such strategies to be futile in reducing their chances of suffering form depression [23]. However, it is not clear why such difference existed for only these two self-help behaviours and only in men, not in women. On the other hand, men who underestimated their risk of developing a MDE were also less likely to “do something they enjoy”. Theoretically, individuals who underestimate their likelihood of developing an illness have also been described as being optimistic about their invulnerability [24]. Due this optimism, these individual’s may not feel the need to take intentional preventative action against their risk of developing major depression.

Among both men and women, individuals who self-rated their overall health status as ‘poor/fair’ had lower self-help scores. We could also view this behaviour from a lens of learned helplessness, in that individuals with poor overall health may view themselves as unable to influence their circumstances, and therefore be less likely to utilize self-help strategies [13]. It is also possible that individuals do not partake in some of these general health strategies because of poor health, preventing them from executing these strategies with ease or independence, such as ‘exercise’ or ‘leaving the house daily’. We found that perceived risk was negatively associated with self-help in women. The higher the risk women perceive themselves to be of developing a MDE, the less likely they are to execute self-help behaviours for their mental health. It is widely recognized that women are at a higher risk of developing an MDE than men [1]. We could attribute this negative relationship between self-help behaviours and risk perception in women (but not men) to feelings of helplessness [13].

In women, the data revealed the most significant relationships between self-help behaviour and women over the age of 50, more-so than women between 31 and 50 yrs., relative to women aged less than 30 yrs. of age. Women of higher ages (65+) were more likely to have higher self-help scores. One possible explanation for this increase in self-help behaviour is an increase in time and independence, which older women may experience in their empty-nest and retirement years [25, 26]. Although the number of dual-income households in Canada has increased over the last several decades, women in heterosexual dual-income households still spend more time on household duties and childrearing than their male counterparts [27]. The lifestyle transition that is empty-nesting, in which the last children leave the house to gain their independence, leaves women (who are statistically more likely to contribute more heavily to their care) with more time to rediscover their identity, and partake in activities and hobbies which they previously may not have had time for [25–27]. In a descriptive study on the effect of retirement of women’s wellbeing, women cited that retirement allowed them to make deliberate improvements in their physical health and diet [25].

It was also seen that women who had attained post-secondary levels of education were more likely to have higher self-help scores. A study by Park and colleagues on the effects of education on health behaviours and beliefs found that individuals with at least a college degree, as compared to those without a college degree, were more likely to have higher levels of health literacy [28]. Individuals who attain higher education may have developed skills such as the ability to research health-related topics, as well as the comprehension skills to understand the complex health issues [29, 30]. It is also possible that higher education may influence psychosocial skills as well. Not only does higher education often result in larger social circles (for social support), but Park and colleagues found that individuals with higher educational attainment were more likely to believe that health behaviours can help in illness prevention [28]. This increase in the belief of an internal locus of control is particularly important in terms of depressive disorders, in which individuals may develop a sense of helplessness and lack of self-efficacy [28, 13]. It is currently unclear as to why this relationship was seen only in women and not men.

The implications of this study are firstly that it reinforces the notions that men and women may have a difference in underlying factors which promotes the use of self-help behaviours in regards to mental health. This further emphasizes that the promotion of self-help behaviours as a valid treatment supplement for MDE may require different delivery based on sex, as well as underlying sociodemographic factors such as education and employment status [31]. We also saw that one’s risk of a MDE as perceived by high-risk individuals, and the accuracy of this perception, have the potential to influence which self-help behaviours they use, or do not use. By promoting overall mental health literacy and the role of self-help strategies for individuals at high-risk of developing a MDE, it may be possible to aid these individuals in the utilization of the valid, evidence-based, self-help strategies and improve long-term health outcomes [32, 6]. Further research is required in order to support the findings of this study and their potential implications. The accuracy of risk perception is the result of comparing perceived and objective risk. Both perceived and objective risks are new in the realm of mental health [8]. In the Health Belief Model, perceived risk is a prominent construct influencing one’s health behaviours [8]. Given the fast growing field of risk predictive analytics, research is needed to investigate the potential effect of objective risk on health behaviours related to mental health, if one’s perceived risk may be changed, and how enhanced accuracy of risk perception may motivate individuals to take preventive actions.

The study has several limitations. Firstly, this study was conducted in Canada and therefore caution should be given in interpreting and applying the findings of this study to other geographic locations. Secondly, the definitions regarding risk-perception accuracy groups (accurate, over-estimator, under-estimator) were arbitrary and based on the methods of Rimer et al. [19] and Lerman et al. [20] This method may be associated with misclassification bias in the relatively low-risk category, however only 3% of participants rated themselves as 0% risk of developing a MDE in the next 4 years. Thirdly, our data were based on self-report and therefore could be subject to recall and reporting bias, especially since some of the survey topics could be subject to self-stigma regarding mental health [33]. This was a cross-sectional study and therefore temporality and causal inference cannot be made. Finally, the participants were at high risk of MDE and therefore caution should be taken when extrapolating the results to low risk populations.

## Conclusion

This is one of the first studies to examine the relationship between self-help behaviour and risk perception in individuals at high-risk for developing major depression. We found sex differences and similarities in the factors associated with self-help. The results of this study can inform future research in mental health education and promotion, as well as risk communication. More research is needed to replicate the results of this study and identify strategies for promoting effective self-help as a means of early prevention of depression.

## Data Availability

The datasets used and/or analysed during the current study are available from the corresponding author on reasonable request.

## Abbreviations

MDD: Major depressive disorder
MDE: Major depressive episode
RCT: Randomized-control trial
MVRPs: Multivariate risk predictors
SSUS: Self-help Management Strategy Use Scale
GAF: Global Assessment of Functioning
DSM-IV: Diagnostic and Statistic Manual of Mental Disorders: Fourth Edition
